# Selective and Catalyst-free Oxidation of D-Glucose to D-Glucuronic acid induced by High-Frequency Ultrasound

**DOI:** 10.1038/srep40650

**Published:** 2017-01-13

**Authors:** Prince N. Amaniampong, Ayman Karam, Quang Thang Trinh, Kai Xu, Hajime Hirao, François Jérôme, Gregory Chatel

**Affiliations:** 1 INCREASE (FR CNRS 3707), ENSIP, 1 rue Marcel Doré, TSA41105, 86073 Poitiers Cedex 9, France; 2 Institut de Chimie des Milieux et Matériaux de Poitiers (IC2MP), Université de Poitiers, CNRS, ENSIP, 1 rue Marcel Doré, TSA41105, 86073 Poitiers Cedex 9, France; 3 Cambridge Centre for Advanced Research and Education in Singapore (CARES), Nanyang Technological University, 1 Create Way, 138602, Singapore; 4 Division of Chemistry and Biological Chemistry, School of Physical and Mathematical Sciences, Nanyang Technological University, 21 Nanyang Link, 637371, Singapore

## Abstract

This systematic experimental investigation reveals that high-frequency ultrasound irradiation (550 kHz) induced oxidation of D-glucose to glucuronic acid in excellent yield without assistance of any (bio)catalyst. Oxidation is induced thanks to the *in situ* production of radical species in water. Experiments show that the dissolved gases play an important role in governing the nature of generated radical species and thus the selectivity for glucuronic acid. Importantly, this process yields glucuronic acid instead of glucuronate salt typically obtained *via* conventional (bio)catalyst routes, which is of huge interest in respect of downstream processing. Investigations using disaccharides revealed that radicals generated by high frequency ultrasound were also capable of promoting tandem hydrolysis/oxidation reactions.

The necessity to diversify our resources and the general trend of reducing our CO_2_ footprint have opened opportunities to produce chemicals, materials and fuels from renewable feedstocks. In this context, the sugar platform is now the subject of intense investigations. Despite the fact that many promising routes to valuable bio-based chemicals and fuels have been reported through (bio)catalytic conversion or fermentation of sugars, the industrial deployment of these pathways is often hampered by a lack of productivity and a costly downstream processing. Clearly, the search of novel technologies has become a priority in this field^1^.

Oxidation of D-glucose to glucuronic acid is a typical example. Glucuronic acid is a highly valuable chemical and one of the organic building blocks of hyaluronic acid whose current worldwide market is estimated to be over $1 billion^2^. Glucuronic acid is widely used in pharmaceutical and medicinal chemistry for the synthesis of modified drugs. It is also largely used as an additive in the food industry, as an active compound in the pharmaceutical industry particularly for the preparation of skin-care products, as an antioxidant, and as a precursor for the biosynthesis of ascorbic acid (Vitamin C)^3^. Furthermore, it is an intermediate for the production of L-gulonic acid and 6-amino-L-gulonic acid, an interesting building block for the synthesis of fine chemicals, polymers and surfactants^4^.

Nowadays, the synthesis of D-glucuronic acid is achieved *via* the enzymatic oxidation of D-glucose at the C6 position by *Ustulina deusta* bacteria and *bacterium industrium var*. *Hoshigaki* under aerobic conditions^4^. Microbe separation, low process productivity and selectivity, as well as the disposal/recycling of waste water, are the main current drawbacks. Solid catalysts have also been explored for this reaction. Nonetheless, to date, from D-glucose, noble metal catalysts exhibit very low selectivity for D-glucuronic acid. Supported metal catalysts like platinum or palladium dispersed over carbon indeed preferentially lead to the oxidation of the C1 position of glucose, resulting in the formation of D-gluconic acid^5^. D-glucuronic acid was synthesized under air from protected D-glucose (1,2 isopropylidene-D-glucose) in 43% yield over a platinum-carbon based catalyst^6^. Very recently, Wojcieszak *et al*. reported the direct oxidation of D-glucose to glucuronic acid in 53% yield over cesium-promoted gold nanoparticles^7^. Low selectivity and productivity, metal leaching and poor catalyst stability still represent important problems to be overcome for industrial deployment. Electrochemical routes were also reported^8^,^9^. These reactions however need pH-controlled conditions, lead to limited yields of glucuronic acid (2%) and require costly metal (Au or Cu) as electrodes. It is worth mentioning that, in most of the above-cited examples, glucuronic acid was obtained as a carboxylate salt, thus requiring a post-neutralization step prior to processing. Although glucuronic acid clearly exhibits high market potential, its availability remains nowadays limited.

Here, we propose a radically different approach based on high-frequency ultrasound (550 kHz) to efficiently oxidize glucose to glucuronic acid in water and with an unprecedented selectivity. This oxidative route proceeds at 25 °C, is highly selective for glucuronic acid and does not need assistance of any (bio)catalyst, thus considerably simplifying downstream processing. The cavitation induced by ultrasound in water (*i.e*., the formation, growth, and collapse of gaseous microbubbles in the liquid phase) creates locally high pressures (up to 1,000 bar) and temperatures (up to 5,000 K)^10^. At high frequencies (150 to 2,000 kHz), these extreme conditions lead to the formation of radicals^11^, and this technology is currently developed for waste water treatment on an industrial scale in combination with different advanced oxidation processes^12^. Low-frequency ultrasound (20–80 kHz) was explored for glucose oxidation^13^. At low frequency, mostly physical effects (shockwaves, microjets, micro-convection, etc.) occur and oxidation of D-glucose (to gluconic acid) is thus always achieved in combination with an oxidant such as NaOCl/TEMPO/NaBr^13^,^14^,^15^ or H_2_O_2_/iron salt (sono-Fenton process)^16^.

## Results

### Reactor Characterization and Optimization

In this study, oxidation of D-glucose to glucuronic acid was carried out in a 250 mL high-frequency ultrasonic reactor (SinapTec Ultrasonic Technology, NextGen Lab 1000, 550 kHz, designed cylindrical stain steel reactor in a cup-horn configuration with a 75 mm diameter base-surface, and equipped with a cooling system). Prior to testing the high-frequency ultrasonic reactor in the oxidation of glucose, we determined the optimal ultrasonic conditions for the generation of HO^·^ radicals in water by studying the effect of the reaction temperature, the mechanical stirring, the reactor filling and the electric/acoustic powers. The concentration of generated radicals was determined by a KI dosimetry method^17^, and the acoustic power delivered in the medium was determined by calorimetry^18^.

**Figure 1:**
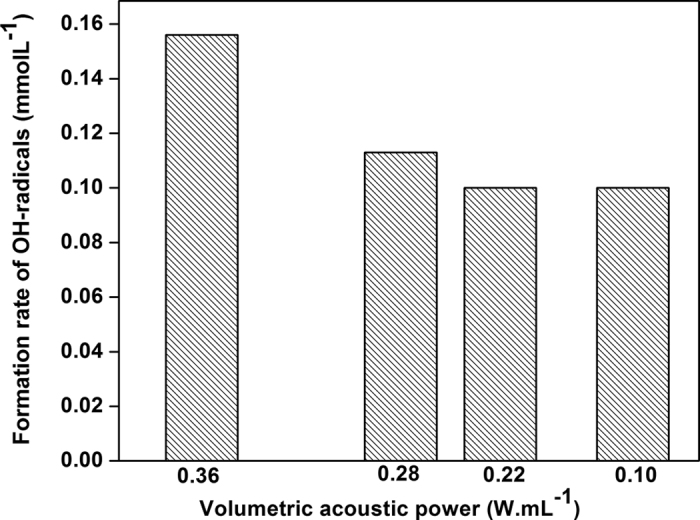
Measured concentration of HO^·^ radicals (mmol.L^−1^) (0.1 M aqueous solution of KI (100 mL), 25 °C, 100 rpm stirring, ultrasonic irradiation (550 kHz, P_acous_ = 0.36 W.mL^−1^, 1 h)).

When the volume of water was changed from 100 to 150, and then to 200 mL, the concentration of radicals was the highest (0.11 mmol.L^−1^) at 100 mL (Figure S1). The concentration of radicals decreased when the amount of water was concomitantly increased over 100 mL. The amount of water indeed has a direct effect on the formation of standing waves during sonication. A large volume of water notably increases the formation of standing waves, which impedes cavitation phenomena and thus reduces the amount of radical species formed in the reactor. The temperature of the reactor (25–55 °C) has no appreciable impact on the concentration of produced radicals (Figure S2). Hence, for practical reasons, ultrasonic reactions were conducted at 25 °C in the following experiments. In contrast to the effect of temperature, the mechanical stirring markedly influences the concentration of radicals. The production rate of radicals was about two times higher at 100 rpm than at 300 rpm (Figure S3). The acoustic power also impacts cavitation phenomena and thus the concentration of *in situ* produced radicals. At 60 W, 60% of the electric energy was converted to acoustic energy (Figure S4). In the optimized configuration of our reactor (25 °C, 100 rpm, 100 mL, 60 W), the acoustic power thus reached a maximum value of 36 W (0.36 W.mL^−1^) for 80% amplitude of the used generator (Figure S5). At this value, the concentration of radicals increased and reached a value close to 0.16 mol.L^−1^ (Fig. 1).

## Discussion

**Figure 2:**
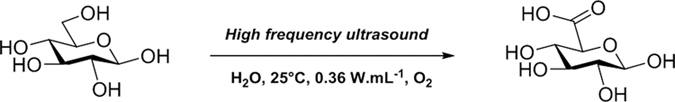
High-frequency ultrasound based glucose oxidation into glucuronic acid.

**Figure 3:**
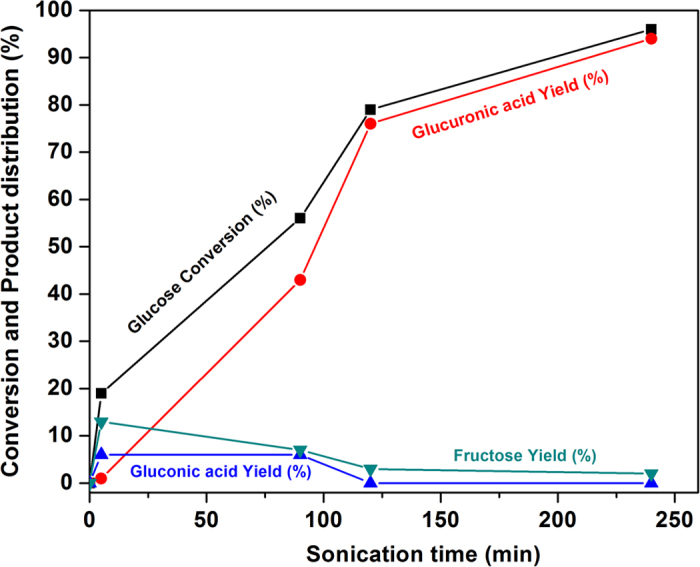
Kinetic profile of the glucose oxidation under high frequency ultrasound (2 g of glucose in 100 mL of H_2_O, 25 °C, 100 rpm stirring, ultrasonic irradiation (550 kHz, P_acous_ = 0.36 W.mL^−1^).

Having in our hands the ideal conditions in term of sonochemical efficiency, the oxidation of glucose with high frequency ultrasound was then investigated (Fig. 2). Typically, 2.0 g of glucose dissolved in 100 mL of distilled water was subjected to an ultrasonic irradiation at a controlled temperature of 25 °C, at a stirring rate of 100 rpm and at a volumetric acoustic power of 0.36 W.mL^−1^. Molecular oxygen was bubbled into the solution at a flow of 10 mL.min^−1^ during sonication. Conversion, yield and selectivity values were determined by HPLC analyses. The kinetic profile is presented in Fig. 3.

Interestingly, at a glucose conversion lower than 20%, fructose and gluconic acid were dominantly produced. When the sonication time was prolonged, fructose and gluconic acid were consumed and glucuronic acid started to be produced, suggesting that fructose and gluconic acid were possible intermediates in the reaction pathway. A mechanistic insight into the reaction is provided later in the manuscript. To our delight, after 4 h of sonication (Fig. 3), the conversion of glucose reached 96% and an unprecedented selectivity of glucuronic acid was observed (98% selectivity, *i.e*. 94% yield). This result constitutes a unique and important advance in the field. In order to support the great selectivity of the process, the aqueous solution was freeze-dried at the end of the reaction and the recovered white solid was analyzed by ^1^H and ^13^C NMR (Figures S6 and S7, respectively). NMR investigations unambiguously confirmed the formation of glucuronic acid as an exclusive product, and the spectra rigorously coincided with those of glucuronic acid standard. In the ^1^H NMR spectrum, the α and β isomers were clearly distinguished and co-existed in a α/β = 1.2 ratio either in D_2_O or in d6-DMSO. In addition, the presence of a broad peak located at 12.74 ppm was also consistent with the presence of a –COOH group. The ^13^C NMR also confirmed the formation of glucuronic acid in the form of two isomers α and β. Another product, in the form of a single isomer, was however observed in the ^13^C NMR spectrum and may be ascribed to the intramolecular esterification of glucuronic acid. Whereas HPLC did not reveal the presence of any impurity during the sonication process, after freeze-drying, formation of a new product was clearly observed, suggesting that this intramolecular esterification did not occur during the sonication process, but during the freeze-drying process. This result is logically understandable because the oxidation of glucose to glucuronic acid was conducted in water.

The crude product was also analyzed by electrospray ionization mass spectroscopy (ESI-MS), and a peak was observed at m/z = 217, which corresponded to the sodium adduct of glucuronic acid (Figure S8). Pure glucuronic acid has exactly the same signature in ESI-MS. Finally, Fourier transform infrared spectroscopy (FTIR) (Figure S9) further confirmed the oxidation of glucose with the appearance of a peak at 1723 cm^−1^ in accordance with the formation of a C=O group. O–H and C–H stretching bands were also clearly visible at 3262 and 2920 cm^−1^, respectively.

**Figure 4:**
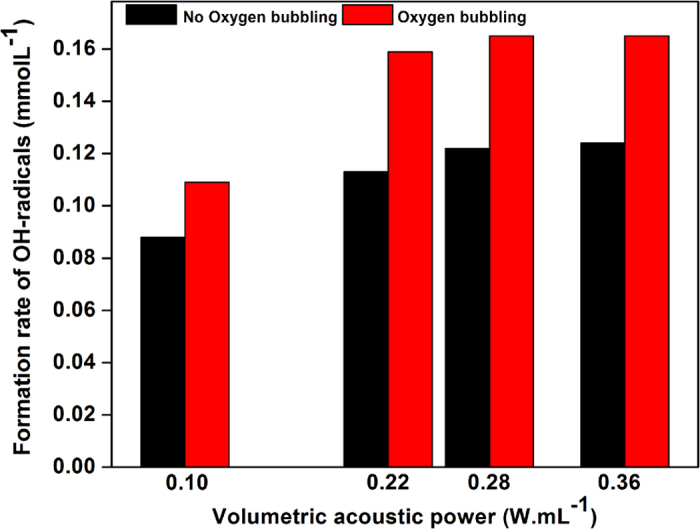
Concentration of HO^·^ radicals as a function of the acoustic power under O_2_ bubbling (10 mL.min^−1^) (0.1 M aqueous solution of KI (100 mL), 25 °C, 100 rpm stirring, ultrasonic irradiation (550 kHz, P_acous_ = 0.36 W.mL^−1^, 1 h)).

To gain further mechanistic insight into the reaction, a few additional experiments were carried out. First of all, when the reaction temperature was increased from 25 °C to 80 °C, the conversion rate of glucose was not changed, which was consistent with a radical mechanism. The selectivity was however markedly reduced from 96% (at 25 °C) to only 55% (at 80 °C) mostly due to the side formation of formic acid at 80 °C (over-oxidation of carbohydrates) (Table 1, Entry 1). When the reaction was conducted without any bubbling of O_2_, *i.e*. in a non-oxygenated aqueous solution of glucose, the conversion rate of glucose was two times lower (Table 1, Entry 2). One may suspect that the bubbling of O_2_ increases the concentration of oxygenated radicals in water, which may rationalize the enhancement of the reaction rate. This hypothesis was further supported by the measurement of the concentration of oxygenated radicals produced in water with or without bubbling of O_2_ (Fig. 4). Whatever the sonication time, the amount of radicals was about 20% larger when O_2_ was bubbled into the solution.

Surprisingly, when the reaction was conducted under an argon atmosphere, 80% of glucose was still converted at 25 °C but the selectivity was markedly different since gluconic acid was the main product (40%) in this case, instead of glucuronic acid in the presence of O_2_ (Table 1, Entry 5). Other detected products were fructose (16% yield) and uronic acid (8% yield). D-arabino-hexos-2-ulose and D-xylo-hexos-4- and 5-ulose were also detected as traces (not quantified). It should be noted that no reaction took place under an O_2_ atmosphere in the absence of ultrasound (Table 1, Entry 6). This difference of selectivity might be ascribed to oxidative species of different nature, and thus of different reactivities, which may have been formed when the ultrasonic process was conducted under O_2_ or Ar. Considering that gluconic acid was observed at the initial stage of the reaction (Fig. 2) and then disappeared as the reaction time was prolonged, one may suggest that the bubbling of O_2_ under ultrasound conditions also facilitated the conversion of gluconic to uronic acid. In this case, oxidation of gluconic acid should provide guluronic acid, a diasteroisomer of glucuronic acid, which was unfortunately not distinctly separated by our HPLC system. To further support this claim, an aqueous solution of gluconic acid was subjected to ultrasonic treatment with a bubbling of O_2_. Under these conditions, 43% of gluconic acid was converted after 120 min of reaction, and an uronic acid was formed in 37% yield (*i.e*. 86% selectivity). It was however difficult to characterize with accuracy the structure of the produced uronic acid (which is expected to be guluronic acid) during the reaction by either ^1^H or ^13^C NMR due to the overlap of different peaks. The reaction was indeed less selective from gluconic acid than from glucose. A distinct signal that is found at 4.3 ppm (-C*H*_2_- in the α of the CO_2_H group) and is characteristic of guluronic acid was however clearly observed. Furthermore, apparition of a peak at the same retention time as that of glucuronic acid was clearly observed in our HPLC system, which strongly supports the formation of an uronic acid from gluconic acid. We thus hypothesize that two parallel reaction pathways occur during the sonication process: (1) the oxidation of glucose to glucuronic acid, which is the largely dominant route and (2) the oxidation of glucose to gluconic and then guluronic acid, which is a very slow process. At this stage, complete elucidation of the reaction mechanism clearly deserves deeper investigations since the nature and amount of dissolved gases may also impact the reaction mechanism in a sensitive manner. For instance, hot-spot temperatures inside the cavitation bubbles have been shown to vary according to the nature of the gas and may also impact the reaction selectivity^19^.

**Figure 5:**
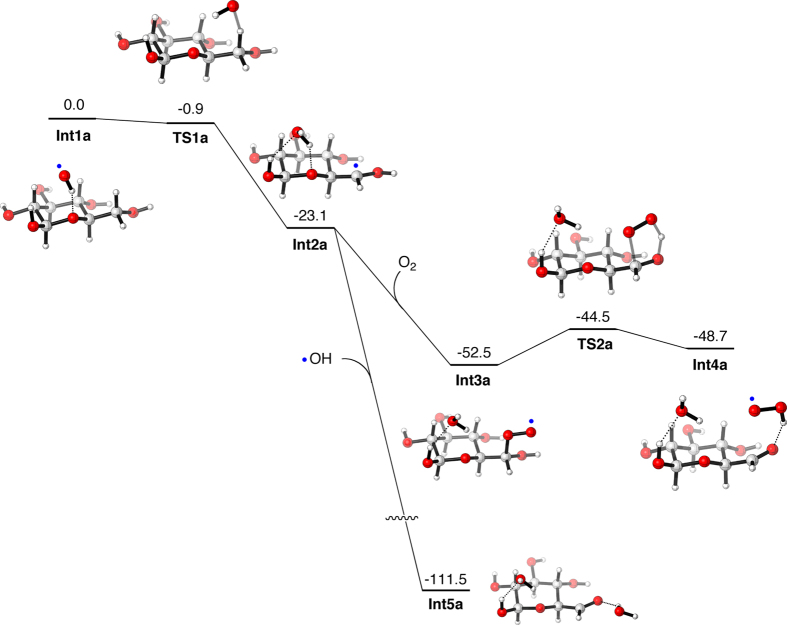
Enthapy profiles (in kcal mol^−1^) for the formation of an aldehyde species in (a) path a and (b) path b. Unpaired electrons of intermediate species are designated by blue dots.

To gain microscopic insights into the possible role of hydroxyl radical generated from high-frequency ultrasound irradiations on the oxidation of D-glucose to D-glucuronic acid, we performed density functional theory (DFT) calculations of the potential energy surfaces at the B3LYP(SCRF)/def2-TZVP//B3LYP/def2-TZVP level, using Gaussian 09^20^,^21^,^22^,^23^,^24^,^25^. The default SCRF method was used to take into account the solvent effect of water. The enthalpy corrections obtained by frequency calculations were added to the potential energy values, to determine enthalpy profiles of reactions. Using the DFT-calculated enthalpy values, we examined whether D-glucuronic acid can be formed when a radical reacts with the hydroxymethyl group of D-glucose. In particular, we examined a pathway in which hydroxy and hydroperoxy radicals abstract hydrogen from the CH_2_ moiety (Fig. 5).

Calculations show that there is no barrier for the first H-abstraction process (**Int1a** to **Int2a**). It should be noted that there is a tiny barrier for this step on the potential energy surface calculated at the B3LYP/def2-TZVP level in the gas phase, but the barrier vanishes after inclusion of the solvent effect and the enthalpy correction (Table S1). The radical species generated as a result of H-abstraction may combine with an O_2_ molecule, if it is present in the system, to form a peroxy radical (**Int2a** to **Int3a**). Subsequently, the distal oxygen of the peroxy moiety abstracts the hydrogen atom of the hydroxyl group, with an energy barrier of 8.0 kcal mol^−1^ (**Int3a** to **Int4a**), to form an aldehyde species. Alternatively, **Int2a** may react with an OH radical to form the aldehyde species. In either case, the aldehyde formation is rather facile. Calculations also suggest that the same aldehyde species can be formed when the initial H-abstraction occurs at the OH group of the hydroxymethyl group (Figure S10 and Table S2).

**Figure 6:**
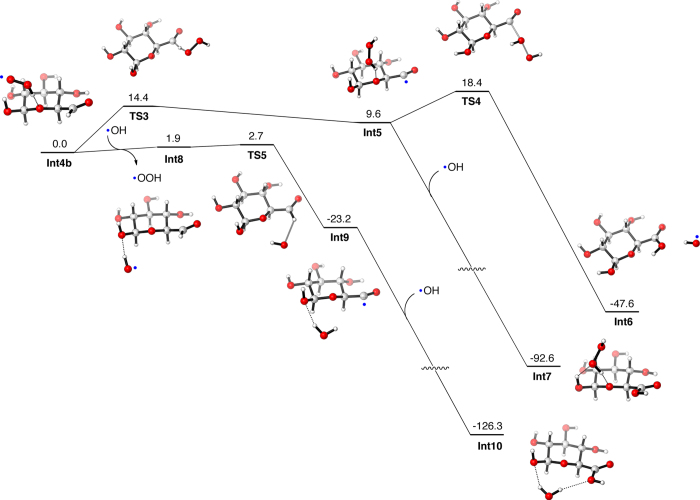
Enthapy profiles (in kcal mol^−1^) for the formation of glucuronic acid from the precursor aldehyde species. Unpaired electrons of intermediate species are designated by blue dots.

The conversion of the aldehyde species to glucuronic acid is also facile, especially when several OH radicals are available for the reaction (Fig. 6). Thus, the formyl hydrogen can be abstracted by an OH radical with a small barrier (2.7 kcal mol^−1^, **Int4b** to **Int9**), and the resultant radical species can combine with another OH radical to form glucuronic acid (**Int9** to **Int10**). Alternatively, the formyl hydrogen may be abstracted by a hydroperoxy radical, but the barrier is somewhat higher (14.4 kcal mol^−1^, **Int4b** to **Int5**) in this case. The barrier seems also to be high (8.8 kcal mol^−1^) when the resultant radical species (**Int5**) reacts with hydrogen peroxide to form **Int6**. However, if an OH radical is available, glucuronic acid should be formed easily (**Int5** to **Int7**).

Altogether, these results reveal that the radical conversion of glucose to glucuronic acid is a favorable reaction. The first abstraction of the hydrogen can be carried out either by an OH or a hydroperoxy radical. The conversion of the intermediate aldehyde to the carboxylic group is however more likely to occur in the presence of OH radicals. One should note that the radical hydrogen formed after dissociation of water readily reacts with O_2_ to form a hydroxyperoxy radical which is further converted to H_2_O and O_2_ by recombination with an OH radical^26^.

Next, our promising strategy of using high frequency ultrasonic irradiation to promote the oxidation of sugars was applied to fructose, mannose, maltose and cellobiose to check the scope of the methodology (Table 2). Reaction conditions similar to those described above for glucose were applied to these sugars. From monosaccharides such as fructose and mannose (Table 2, Entries 1 and 2), the corresponding uronic acids were produced in 54% and 65% yields, respectively. Other products were also detected, particularly glucose (stemming from isomerization reactions) and hexonic acids. In addition, xylose was detected presumably due to an over-oxidation of hexoses as corroborated by the detection of formic acid in this case.

Importantly, glucuronic acid can also be produced from cellobiose, the monomer of cellulose (Table 2, Entry 3). To our delight, after 2 h of irradiation in the ultrasonic reactor, 45% of cellobiose was converted to glucuronic acid (7% yield), glucose (27% yield) and gluconic acid (11% yield). This result indicates that high-frequency ultrasound also promotes the tandem radical hydrolysis/oxidation of cellobiose, an important perspective for the production of glucuronic acid directly from lignocellulosic biomass (out of the scope of this paper). Other detected products were maltose and arabinose stemming from isomerization and over-oxidation reactions.

As with cellobiose, maltose was converted smoothly to its corresponding uronic acid with 76% selectivity, further confirming that hydroxyl radical generated in the high frequency ultrasonic system also promotes the cleavage of the glycosidic bond (Table 2, Entry 4).

We would like to point out that the optimized conditions for glucose might not represent the optimized conditions for all the other tested carbohydrates in Table 2, since the structure of saccharides directly affects the reactivity with radical species. Nevertheless, the results collected in Table 2 clearly highlight the potential of high frequency sonochemistry for the selective and catalyst-free oxidation of sugars.

In conclusion, we have reported that high-frequency ultrasound treatment is capable of oxidizing D-glucose to glucuronic acid selectively in water at ambient temperature and without the aid of any catalyst, which should therefore facilitate downstream processing. Importantly, not only is this work much more selective than current (bio)catalytic routes, but it also provides glucuronic acid instead of glucuronate salt that is formed with common (bio)routes. Employing different atmospheres, the reaction product can be tuned either toward gluconic (under Ar) or toward glucuronic acid (under O_2_). The process is also applicable to disaccharides such as cellobiose, thus offering an interesting perspective of this work particularly for the synthesis of glucuronic acid from non-edible cellulosic biomass (under investigations in our group). Noteworthy, although out of the scope of this present study, the unique role of radicals generated by high frequency ultrasound in promoting isomerization reactions also presents an interesting perspective for carbohydrate processing.

Future work will be directed to sonication of more concentrated feed of glucose not only to reduce the energy consumption per g of treated glucose, but also to increase the productivity of the whole process.

## Methods

### Carbohydrates oxidation

Oxidation of glucose, fructose, mannose, cellobiose and maltose was carried out in a 250 mL high-frequency ultrasonic reactor (SinapTec Ultrasonic Technology, NextGen Lab 1000). Typically, 2.0 g of glucose in 100 mL of distilled water was subjected to high-frequency ultrasound irradiation at a controlled temperature of 25 °C and a frequency of 550 kHz (standby power P_0_ = 13.9 W, nominal electric power of the generator P_elec_ = 46.1 W), with an acoustic power in water of P_acous.vol_ = 0.36 W.mL determined by calorimetry using the procedure described in the literature^18^. Energy consumption was measured by using a wattmeter (Perel^®^).

### Product analysis

Liquid products (glucose, fructose, maltose, mannose and cellobiose) were analyzed using a Shimadzu HPLC equipped with a pump system (LC-20AD), autosampler SIL-10A, and controller CBM 20 A. Products were separated by using a Shodex Sugar KS800 × 8.0 mm (size-exclusion chromatography; SEC) column using ultrapure water as eluent and a flow rate of 1.0 mL.min^−1^, and quantified by a refractive index detector (Shimadzu RID-10A). The amounts of glucuronic acid and other acid products were determined from the HPLC analysis by using a Varian Pro Star HPLC equipped with an ICE-COREGEL 107 H column 300 × 7.8 mm from Transgenomic, a UV/Vis detector (Varian Pro Star, 210 nm) and a refractive index detector (Varian 356-LC). A H_2_SO_4_ aqueous solution was used as the eluent with 0.4 mL.min^−1^ flow rate. External calibration of liquid chromatography was performed using standards of glucose, fructose, maltose, mannose and cellobiose, and glucuronic acid was quantified by the difference between the two HPLC analyses. For GC-MS analysis, liquid products were lyophilized and silylated before injection. For the protocol of silylation: 10 mg of sample was dissolved in 1 mL of pyridine (1 mg sorbitol/1 mL pyridine), followed by the sequential addition of 500 μL of HMDS and 500 μL of chlorotrimethylsilane. A white precipitation was obtained, and before injection the silylated sample was centrifuged at 8000 rpm for 10 min.

Potassium iodide dosimetry was performed as described in the literature, with 0.1 mol.L^−1^ KI solution over 30 min^17^. I_3_ formation was monitored by using a UV/Vis spectrophotometer (Evolution 60 S from Thermo Scientific) at a wavelength of 355 nm (ε (I_3_^−^) = 26,303 L.mol^−1^.cm^−1^). Each experiment was repeated three times at 22.5 °C, which was maintained by using a Minichiller cooler (Huber).

## Additional Information

**How to cite this article**: Amaniampong, P. N. *et al*. Selective and Catalyst-free Oxidation of D-Glucose to D-Glucuronic acid induced by High-Frequency Ultrasound. *Sci. Rep.* **7**, 40650; doi: 10.1038/srep40650 (2017).

**Publisher's note:** Springer Nature remains neutral with regard to jurisdictional claims in published maps and institutional affiliations.

## Figures and Tables

**Table 1:** Effect of high-frequency ultrasonic irradiation on glucose solution and observed products at different substrate conditions.

Entry	Time (min)	Conv. (%)	Glucuronic acid yield (%)	Fructose yield (%)	Gluconic yield (%)	Formic acid yield (%)	Cellobiose yield (%)	Selectivity to glucuronic acid (%)
1	120[a,b]	80	44	<1	7	28	—	55
2	5	9	<1	6	2	—	—	—
3	5[a]	19	<1	13	6	—	—	—
4	240[a]	96	94	2	—	—	—	98
5	120[c]	80	8	16	40	—	—	10
6	120[d]	—	—	—	—	—	—	—

Experimental conditions: 2 g of glucose in 100 mL of H_2_O, 25 °C, 100 rpm stirring, ultrasonic irradiation (550 kHz, P_acous_ = 0.36 W.mL^−1^).

^[a]^Glucose solution was oxygenated at a rate of 10 mL.min^−1^.

^[b]^Reaction temperature of 80 °C.

^[c]^Glucose solution was deoxygenated by bubbling argon at a rate of 10 mL.min^−1^ (other reaction products detected include D-arabino-hexos-2-ulose, D-xylo-hexos-4-and 5-ulose).

^[d]^Blank reaction in the absence of ultrasonic irradiation.

**Table 2:** Synthesis of corresponding uronic acid from different carbohydrates.

Entry	Carbohydrate	Conv. (%)	Yield to corresponding uronic acid (%)	Yield to glucose (%)	Yield to corresponding hexonic acid (%)
1	Fructose	91	54	10	20
2	Mannose	90	65	6	18
3	Cellobiose	45	7	27	11
4	Maltose	25	19	5	<2

Experimental conditions: 20 g.L^−1^ concentration of substrates, 10 mL.min^−1^ O_2_ flow rate, 100 mL volume of H_2_O, 120 min sonication time (550 kHz, P_acous_ = 0.36 W.mL^−1^). Other products detected include formic acid and xylose.

## References

[b1] Jerome F., Chatel G. & De Oliveira Vigier K. Depolymerization of cellulose to processable glucans by non-thermal technologies. Green Chemistry, Green Chem. 18, 3903–3913 (2016).

[b2] Liu L., Liu Y., Li J., Du G. & Chen J. Microbial production of hyaluronic acid: current state, challenges, and perspectives. Microbial cell factories 10, 1 (2011). 22088095 10.1186/1475-2859-10-99 PMC3239841

[b3] Michal G. & Schomburg D. Biochemical pathways: an atlas of biochemistry and molecular biology. (Wiley New York, 1999).

[b4] Röper H. Selective Oxidation of D‐Glucose: Chiral Intermediates for Industrial Utilization. Starch‐Stärke 42, 342–349 (1990).

[b5] Abbadi A. & Van Bekkum H. Effect of pH in the Pt-catalyzed oxidation of D-glucose to D-gluconic acid. Journal of Molecular Catalysis A: Chemical 97, 111–118 (1995).

[b6] Mehltretter C., Alexander B., Mellies R. & Rist C. A Practical Synthesis of D-Glucuronic Acid through the Catalytic Oxidation of 1, 2-Isopropylidene-D-glucose2. Journal of the American Chemical Society 73, 2424–2427 (1951).

[b7] Wojcieszak R., Cuccovia I. M., Silva M. A. & Rossi L. M. Selective oxidation of glucose to glucuronic acid by cesium-promoted gold nanoparticle catalyst. Journal of Molecular Catalysis A: Chemical (2016).

[b8] Marioli J. M. & Kuwana T. Electrochemical characterization of carbohydrate oxidation at copper electrodes. Electrochimica Acta 37, 1187–1197 (1992).

[b9] Torto N. Recent progress in electrochemical oxidation of saccharides at gold and copper electrodes in alkaline solutions. Bioelectrochemistry 76, 195–200 (2009). 19617004 10.1016/j.bioelechem.2009.06.009

[b10] Cintas P. & Luche J.-L. Green chemistry. The sonochemical approach. Green Chemistry 1, 115–125 (1999).

[b11] Mason T. J. & Peters D. Practical sonochemistry: Power ultrasound uses and applications. (Woodhead Publishing, 2002).

[b12] Mahamuni N. N. & Adewuyi Y. G. Advanced oxidation processes (AOPs) involving ultrasound for waste water treatment: a review with emphasis on cost estimation. Ultrasonics Sonochemistry 17, 990–1003 (2010). 19879793 10.1016/j.ultsonch.2009.09.005

[b13] Kardos N. & Luche J.-L. Sonochemistry of carbohydrate compounds. Carbohydrate Research 332, 115–131 (2001). 11434369 10.1016/s0008-6215(01)00081-7

[b14] Brochette-Lemoine S., Joannard D., Descotes G., Bouchu A. & Queneau Y. Sonocatalysis of the TEMPO-mediated oxidation of glucosides. Journal of Molecular Catalysis A: Chemical 150, 31–36 (1999).

[b15] Brochette-Lemoine S. . Ultrasound in carbohydrate chemistry: sonophysical glucose oligomerisation and sonocatalysed sucrose oxidation. Ultrasonics sonochemistry 7, 157–161 (2000). 11062868 10.1016/s1350-4177(99)00035-8

[b16] Rinsant D., Chatel G. & Jérôme F. Efficient and Selective Oxidation of D‐Glucose into Gluconic acid under Low‐Frequency Ultrasonic Irradiation. ChemCatChem 6, 3355–3359 (2014).

[b17] La Rochebrochard d’Auzay S. d., Blais J.-F. & Naffrechoux E. Comparison of characterization methods in high frequency sonochemical reactors of differing configurations. Ultrasonics sonochemistry 17, 547–554 (2010). 19948421 10.1016/j.ultsonch.2009.10.024

[b18] Koda S., Kimura T., Kondo T. & Mitome H. A standard method to calibrate sonochemical efficiency of an individual reaction system. Ultrasonics sonochemistry 10, 149–156 (2003). 12726951 10.1016/S1350-4177(03)00084-1

[b19] Mason T. & Lorimer J. Theory, applications and uses of ultrasound in chemistry. Ellis Harwood Limited, John Wiley: New York (1988).

[b20] Becke A. D. Density‐functional thermochemistry. III. The role of exact exchange. The Journal of chemical physics 98, 5648–5652 (1993).

[b21] Lee C., Yang W. & Parr R. G. Development of the Colle-Salvetti correlation-energy formula into a functional of the electron density. Physical review B 37, 785 (1988). 10.1103/physrevb.37.785 9944570

[b22] Vosko S., Wilk L. & Nusair M. Accurate spin-dependent electron liquid correlation energies for local spin density calculations: a critical analysis. Canadian Journal of physics 58, 1200–1211 (1980).

[b23] Weigend F. & Ahlrichs R. Balanced basis sets of split valence, triple zeta valence and quadruple zeta valence quality for H to Rn: design and assessment of accuracy. Physical Chemistry Chemical Physics 7, 3297–3305 (2005). 16240044 10.1039/b508541a

[b24] Tomasi J., Mennucci B. & Cammi R. Quantum mechanical continuum solvation models. Chemical reviews 105, 2999–3094 (2005). 16092826 10.1021/cr9904009

[b25] Gaussian09, R. A. 1, Frisch M. J., Trucks G. W., Schlegel H. B., Scuseria G. E., Robb M. A., Cheeseman J. R., Scalmani G., Barone V., Mennucci B., Petersson G. A. . Gaussian. *Inc*., *Wallingford CT* (2009).

[b26] Bremner H. D., Burgess E. A. & Chand R. The chemistry of ultrasonic degradation of organic compounds. Current Organic Chemistry 15, 168–177 (2011).

